# Excessive load promotes temporomandibular joint chondrocyte apoptosis via Piezo1/endoplasmic reticulum stress pathway

**DOI:** 10.1111/jcmm.18472

**Published:** 2024-06-06

**Authors:** Xiaohui Wang, Junli Tao, Jianping Zhou, Yi Shu, Jie Xu

**Affiliations:** ^1^ College of Stomatology Chongqing Medical University Chongqing China; ^2^ Chongqing Municipal Key Laboratory of Oral Biomedical Engineering of Higher Education Chongqing China; ^3^ Chongqing Key Laboratory for Oral Diseases and Biomedical Sciences Chongqing China; ^4^ State Key Laboratory of Ultrasound in Medicine and Engineering Chongqing Medical University Chongqing China

**Keywords:** apoptosis, endoplasmic reticulum stress, mechanotransduction, Piezo1, temporomandibular joint

## Abstract

Excessive load on the temporomandibular joint (TMJ) is a significant factor in the development of TMJ osteoarthritis, contributing to cartilage degeneration. The specific mechanism through which excessive load induces TMJ osteoarthritis is not fully understood; however, mechanically‐activated (MA) ion channels play a crucial role. Among these channels, Piezo1 has been identified as a mediator of chondrocyte catabolic responses and is markedly increased in osteoarthritis. Our observations indicate that, under excessive load conditions, endoplasmic reticulum stress in chondrocytes results in apoptosis of the TMJ chondrocytes. Importantly, using the Piezo1 inhibitor GsMTx4 demonstrates its potential to alleviate this condition. Furthermore, Piezo1 mediates endoplasmic reticulum stress in chondrocytes by inducing calcium ion influx. Our research substantiates the role of Piezo1 as a pivotal ion channel in mediating chondrocyte overload. It elucidates the link between excessive load, cell apoptosis, and calcium ion influx through Piezo1. The findings underscore Piezo1 as a key player in the pathogenesis of TMJ osteoarthritis, shedding light on potential therapeutic interventions for this condition.

## 1 INTRODUCTION

Temporomandibular joint osteoarthritis (TMJOA) is a chronic degenerative joint disorder characterized by progressive cartilage degradation, subchondral bone remodelling, synovitis, and chronic pain. TMJOA commonly results from various factors such as chronic joint wear, trauma, disc displacement or dislocation, and autoimmune diseases.^1^, ^2^, ^3^, ^4^

A close relationship exists between excessive loads and TMJOA. Altered mechanical stresses on the joint due to mouth breathing may impact condylar development.^5^ Mechanical pressure alters signalling in chondrocytes, affecting cartilage maintenance, degradation, and regeneration. This plays a crucial role in the pathogenesis of osteoarthritis.^6^, ^7^, ^8^

Mechanically‐activated (MA) ion channels, notably transient receptor potential cation channel subfamily V member 4 (TRPV4) and Piezo, are crucial on the chondrocyte membrane for mechanotransduction mechanisms.^6^ Piezo, a class of mammalian mechanosensitive ion channels identified in 2010, can transduce diverse mechanical stimuli, including pressure, tension, and shear forces, with two subtypes, Piezo1 and Piezo2.^9^, ^10^, ^11^, ^12^ Recent studies indicate that Piezo1 transmits injurious mechanical signals, leading to chondrocyte catabolic metabolic responses.^6^ The expression of Piezo1 is significantly elevated in human osteoarthritic cartilage.^7^, ^13^

When mechanotransduction occurs through mechanically activated ion channels on the cell membrane, mechanical stimuli open these channels, causing significant ion flow and altering the membrane potential. Notably, when this ion flow involves Ca^2+^ ions, the changed concentration of Ca^2+^ inside and outside the cell affects chondrocyte and extracellular matrix (ECM) metabolism. Abnormal intracellular Ca^2+^ levels can lead to cell death. Furthermore, the influx of extracellular Ca^2+^ can cause endoplasmic reticulum calcium overload, leading to the accumulation of unfolded proteins in the ER. This condition ultimately induces endoplasmic reticulum stress (ERS) and apoptosis.^14^, ^15^, ^16^, ^17^ Thus, we hypothesize that certain mechanically activated ion channels, under excessive load, mediate the influx of extracellular Ca^2+^, eventually triggering ERS and apoptosis in chondrocytes.

To examine the impact of overloading pressure on TMJ chondrocytes, it is crucial to replicate the in vivo microenvironment and establish an in vitro mechanical pressure model. TMJ cartilage primarily consists of type I collagen derived from the ECM. Gelatin methacrylate (GelMA) hydrogels have gained widespread use in chondrocyte in vitro cultivation and tissue engineering due to their favourable bioreactivity, biocompatibility, non‐immunogenicity, and tunability.^18^, ^19^, ^20^, ^21^ Additionally, the cartilage matrix is abundant in hyaluronic acid. Copolymerizing a small amount of hyaluronic acid methacrylate (HAMA) significantly enhances chondrocyte redifferentiation, resulting in the formation of transparent, cartilage‐like neo‐tissues with a progressive increase in mechanical strength.^22^, ^23^, ^24^ GelMA/HAMA hydrogels were employed to mimic cartilage, creating a more representative cartilage microenvironment. This approach facilitated the exploration of cellular pathway alterations under pressure.^25^, ^26^, ^27^

In this study, we employed an in vivo forced mouth‐opening model and an ex vivo three‐dimensional culture Flexcell pressure loading system to simulate the conditions of chondrocytes under overload pressure. We identified Piezo1 as a crucial ion channel that is upregulated and activated on the membrane of chondrocytes in response to overload pressure. Additionally, we confirmed that overload pressure results in the influx of calcium ions through Piezo1 into the cells, triggering ERS‐induced apoptosis in chondrocytes. This suggests that early application of gsmtx4 to inhibit Piezo1 may have a suppressive effect on chondrocyte degeneration, providing a novel avenue for the early treatment of temporomandibular joint osteoarthritis.

## 2 MATERIALS AND METHODS

### 2.1 CBCT imaging

Experienced radiologists at Chongqing Medical University Affiliated Stomatological Hospital conducted the CBCT scans, with patients using the same CBCT machine. The baseline during scanning was parallel to the orbit‐nasal passage plane. Patients were instructed to relax their heads naturally, positioning the mandible in retruded contact. The scanning encompassed the region from the upper margin of the orbit to the chin.

### 2.2 Cell culture

Sprague Dawley (SD) rat (males, 4 weeks, *n* = 6). Under aseptic conditions, cartilage was carefully isolated. All cartilage specimens were washed with PBS, cut into small pieces (1–2 mm^2^), and digested in 0.25% trypsin (Mengbio, Chongqing, China) for 10 min, followed by digestion in 2 mg/mL type II collagenase for 1 h. After centrifugation at 1000 rpm for 10 min, chondrocytes were collected. The chondrocytes were suspended in DMEM (Gibco, New York, USA) containing 15% fetal bovine serum (Gibco, New York, USA) and 1% penicillin/streptomycin, then cultured at 37°C in a humidified atmosphere with 5% CO_2_. The culture medium was changed every 3 days.

### 2.3 Three‐dimensional culture of chondrocytes

9.5% Glema (EFL, Suzhou, China) and 0.5% Hama (EFL, Suzhou, China) were dissolved in 0.25% LAP photoinitiator (EFL, Suzhou, China).^18^, ^24^, ^25^, ^28^ Chondrocytes were passaged to P2, digested with trypsin, and after centrifugation, cells were resuspended in a hydrogel mixture with a cell concentration of 1 × 10^7^ cells/mL. The synthesized cell‐hydrogel suspension was added to a polydimethylsiloxane mould and exposed to 405 nm light for 30 s. Subsequently, the synthesized hydrogel chondrocyte constructs were placed in a culture medium, with medium changes every 3 days, and cultured for 14 days.^26^

### 2.4 Mechanical test

The compression modulus of GelMA/HAMA‐chondrocyte hydrogel disks (thickness = 3 mm, diameter = 10 mm) was determined by using a universal testing machine (MSD, New Jersey, USA). At a strain rate of 0.2 mm/min, the stress–strain curve was obtained. The compression modulus was determined by calculating the slope of the stress–strain curve within the linear region in the 0%–20% strain range.^29^

### 2.5 In vitro cell compression

The hydrogel blocks cultured for 14 days were placed into Biopress culture plates (Flexcell International, Burlington, NC, USA) and subjected to compression using the FX‐5000C™ FLEXCELL® Compression Plus™ System (Flexcell International, Burlington, NC, USA) at a sinusoidal wave of 0.5 Hz, 40 kPa for 2 and 6 h, respectively. The hydrogel blocks without applied pressure served as the control group. During compression, GSMTX4 (MedChemExpress, HY‐P1410, New Jersey, USA) and calcium‐free culture medium were used to treat the cell blocks.^30^

### 2.6 Live/dead cell staining

The viability of chondrocytes encapsulated in Glema/Hama hydrogel after compression was assessed through live/dead cell staining. After 0, 2, and 6 h of compression, the Glema/Hama hydrogel was washed twice with PBS and stained with Calcein/PI Cell Viability/Cytotoxicity Assay Kit (Beyotime, C2015, Shanghai, China) for 30 min. Subsequently, the hydrogel was washed three times with PBS and three‐dimensional images were captured using an upright fluorescence microscope (Carl Zeiss, Baden‐Württemberg, Germany).

### 2.7 Quantitative real‐time polymerase chain reaction (qRT‐PCR) analysis

Total RNA was isolated from chondrocytes using RNAiso plus (Takara, Nogihigashi, Japan). Complementary DNA was obtained by using the PrimeScript™ RT reagent kit with a gDNA eraser (Takara Nogihigashi, Japan). Next, Real‐time PCR was performed on a BIORAD real‐time PCR system (CFX Connect, CA, USA) with 40 cycles using Power TB green PCR Master Mix (Takara Nogihigashi, Japan). Target genes were normalized by GAPDH. The expression of target genes was reported using the 2^−ΔΔct^ method. The primer sequences are shown in Table 1.

**TABLE 1:** Rat gene primers.

Gene	Forward primer	Reverse primer
Ddit3	TGGAAGCCTGGTATGAGGATCTG	GAGGTGCTTGTGACCTCTGCTG
Caspase12	CAATTCCGACAAACAGCTGAGTTTA	CATGGGCCACTCCAACATTTAC
Atf6	ATCACCTGCTATTACCAGCTACCAC	TGACCTGACAGTCAATCTGCATC
Hspa5	TCAGCCCACCGTAACAATCAAG	TCCAGTCAGATCAAATGTACCCAGA
Eif2ak3	CCAAGCTGTACATGAGCCCAGA	TTTCTGAGTGAACAGTGGTGGAAAC
Ern1	CATCACCATGTATGACACCAAGACC	TGTCCACAGTTACCACCAGTCCA
Piezo1	CCCTGTCAGTCTACATCAGTTGG	TCTGTGTGGGCTCTGTGTCG
Gapdh	GGCACAGTCAAGGCTGAGAATG	ATGGTGGTGAAGACGCCAGTA

### 2.8 Immunofluorescence staining

The Glema/Hama hydrogel was fixed with 4% paraformaldehyde (Beyotime, Shanghai, China) for 4 h, dehydrated overnight in 30% sucrose, embedded in OCT (optimal cutting temperature) compound, and stored at −80°C overnight. The embedded Glema/Hama hydrogel was sectioned into 20 μm thick specimens. The sections were permeabilized with 0.2% Triton‐100 for 10 min, washed with PBS, and then blocked with normal goat serum at room temperature for 1 h. The sections were then incubated overnight at 4°C with primary antibodies against p‐PERK (Abbkine ABP50528, CA, USA), p‐ERN1 (Abcam ab48187, Cambridge, England), caspase‐12 (Abcam ab62484, Cambridge, England), and CHOP (Proteintech 15,204‐1‐AP, Wuhan, China). After washing with PBS, the sections were incubated with Alexa Fluor555‐conjugated goat anti‐rabbit secondary antibody (CST 4412s, Boston, USA) at room temperature in the dark for 1 h. Finally, the sections were mounted using an anti‐fluorescence quenching mounting medium containing DAPI (Beyotime P0131, Shanghai, China). Image acquisition was performed using an upright fluorescence microscope (Carl Zeiss, Baden‐Württemberg, Germany).

### 2.9 Safranin‐O staining

The deposition of ECM by chondrocytes was assessed using Safranin O staining. The hydrogel was frozen and sectioned and then stained with Safranin O stain (Solarbio, Beijing, China) following the manufacturer's instructions. The staining results were recorded using an inverted microscope.

### 2.10 High‐throughput RNASeq analysis of chondrocyte

The chondrocytes pressed for 0, 2, and 6 h were subjected to high‐throughput RNAseq analysis by Novogene Bioinformatics Institute (Beijing, China) to screen for differentially expressed mRNAs. The differentially expressed protein‐coding genes were subjected to KEGG enrichment analysis using the Kobas software.

### 2.11 Transmission electron microscopy

The chondrocytes treated with the Flexcell system were fixed in 2.5% paraformaldehyde phosphate buffer (0.01 M, pH = 7.4) at 4°C for 24 h. The samples were fixed in a 1% osmium tetroxide solution for 1–2 h, rinsed three times with 0.1 M phosphate buffer (PB) at pH 7.4, and subsequently subjected to graded dehydration using a series of acetone solutions. Following insertion into embedding moulds, the samples were subjected to baking at 60°C in an oven. Using an ultramicrotome (Leica UC7, Wetzlar, Germany), resin blocks were sectioned into 70–90 nm ultrathin slices. The sections were stained with uranyl acetate and lead citrate and observed using a transmission electron microscope (Hitachi‐7800, Tokyo, Japan).

### 2.12 In vivo forced‐mouth opening model

All animal experiments complied with the Animals (Scientific Procedures) Act (1986), and the approval of all procedures was granted by the Ethics Committee of The Affiliated Hospital of Stomatology, Chongqing Medical University [CQHS‐REC‐2023 (LSNo.042)]. SD rats were obtained from Chongqing Enswell Biotechnology Co. They were housed in a clean room with a 12‐h light–dark cycle. SD rats were acclimated in the animal facility for at least 7 days before use and provided with food and water ad libitum. Male SD rats (8 weeks old, 200–250 g) were randomly divided into three groups: 5‐day group (*n* = 18), 10‐day group (*n* = 18), and 20‐day group (*n* = 18). Each group of SD rats was further divided into three subgroups: control group, model group, and treatment group, with six rats in each subgroup. The model group underwent 2 h of daily mouth opening to induce the temporomandibular joint cartilage degeneration, while the treatment group received a total volume of 50 μL of GsMTx4 (medchemexpress, HY‐p1410, New Jersey, USA) through local injection into the TMJ. The model group was treated with physiological saline.^31^, ^32^ At the end of the experiment, TMJ samples were collected. After euthanizing each rat, the left temporomandibular joint was exposed and scanned using microCT to assess the condyle. The right intact joint was fixed in 4% paraformaldehyde for 24 h, decalcified with 19% EDTA, and then embedded in paraffin. Sagittal sections of 4 mm thickness were obtained and subjected to staining.

### 2.13 Safranin O and fast green staining

All paraffin‐embedded temporomandibular joint samples were sectioned into 4 μm slices. SO&FG staining (Solarbio, Beijing, China) was used to detect changes in proteoglycans. Mankin scoring system was used to grade and assess bone destruction, with higher scores indicating more severe degeneration.^33^, ^34^, ^35^

### 2.14 Immunohistochemistry

Immunohistochemistry staining was performed using antibodies against p‐PERK (Abbkin ABP50528, CA, USA), p‐ERN1 (Abcam ab48187, Cambridge, USA), caspase‐12 (Abcam ab62484, Cambridge, USA), and CHOP (Proteintech 15,204‐1‐AP, Wuhan, China). After dewaxing, hydration, and antigen retrieval of the sections, they were incubated with blocking peroxidase (Beijing Zhongshan PV‐6001, Beijing, China) for 10 min, followed by blocking with 10% normal goat serum for 30 min. The primary antibodies were incubated overnight at 4°C. On the following day, after washing the slides with PBS, they were incubated with enzyme‐labelled goat anti‐rabbit polymer at 37°C for 30 min. DAB (Beijing Zhongshan ZLI‐9018, Beijing, China) was used as the substrate for colour development. All slides were counterstained with haematoxylin. The images of histological stained sections were obtained using an Olympus microscope (Olympus, Tokyo, Japan). Image analysis software (ImageJ; Media Cybernetics) was used to measure the average optical density of p‐PERK, p‐ERN1, CHOP, and caspase‐12 in the cartilage.

### 2.15 Statistical analysis

The experiments were conducted independently three times, and differences in variables between groups were assessed with GraphPad 9.0 (GraphPad Software Inc., La Jolla, CA). The significant differences were used by *t*‐test or one‐way anova with Tukey's post hoc test. *p* < 0.05 is regarded as statistically significant. All data were presented as the mean ± SD unless otherwise indicated.

## 3 RESULTS

### 3.1 Mouth breathing can impose excess strain on the temporomandibular joint, potentially resulting in alterations to the condyle

Clinical case reports disclose bilateral TMJ condylar resorption in a 16‐year‐old patient who exhibits mouth breathing. After ruling out factors like trauma, malocclusion, and genetics, the patient received a four‐month corrective treatment for mouth breathing to relieve the temporomandibular joint's load. Subsequent CT results revealed continuous condylar cortical bone and successful condyle reconstruction (Figure S1A,B).

### 3.2 Chondrocytes are cultured in a three‐dimensional environment to replicate the in vivo microenvironment

To replicate the in vivo microenvironment of chondrocytes, we cultured them in a mixture of 9.5% GelMA and 0.5% HAMA (Figure S2A–C). After 14 days, Safranin O staining was performed to observe cell morphology and cartilage matrix production. The results revealed a significant increase in positive protein‐polysaccharide staining, indicating enhanced chondrocyte activity (Figure S2D). We assessed the compressive strength of the hydrogel‐chondrocyte constructs after 14 days using a universal testing machine. The compressive modulus was determined as (22.47 ± 4.96 kPa) when the hydrogel deformation reached 20% (Figure S2E).

### 3.3 Overloading pressure upregulates the expression of ERS‐apoptosis‐related genes in chondrocytes

**FIGURE 1:**
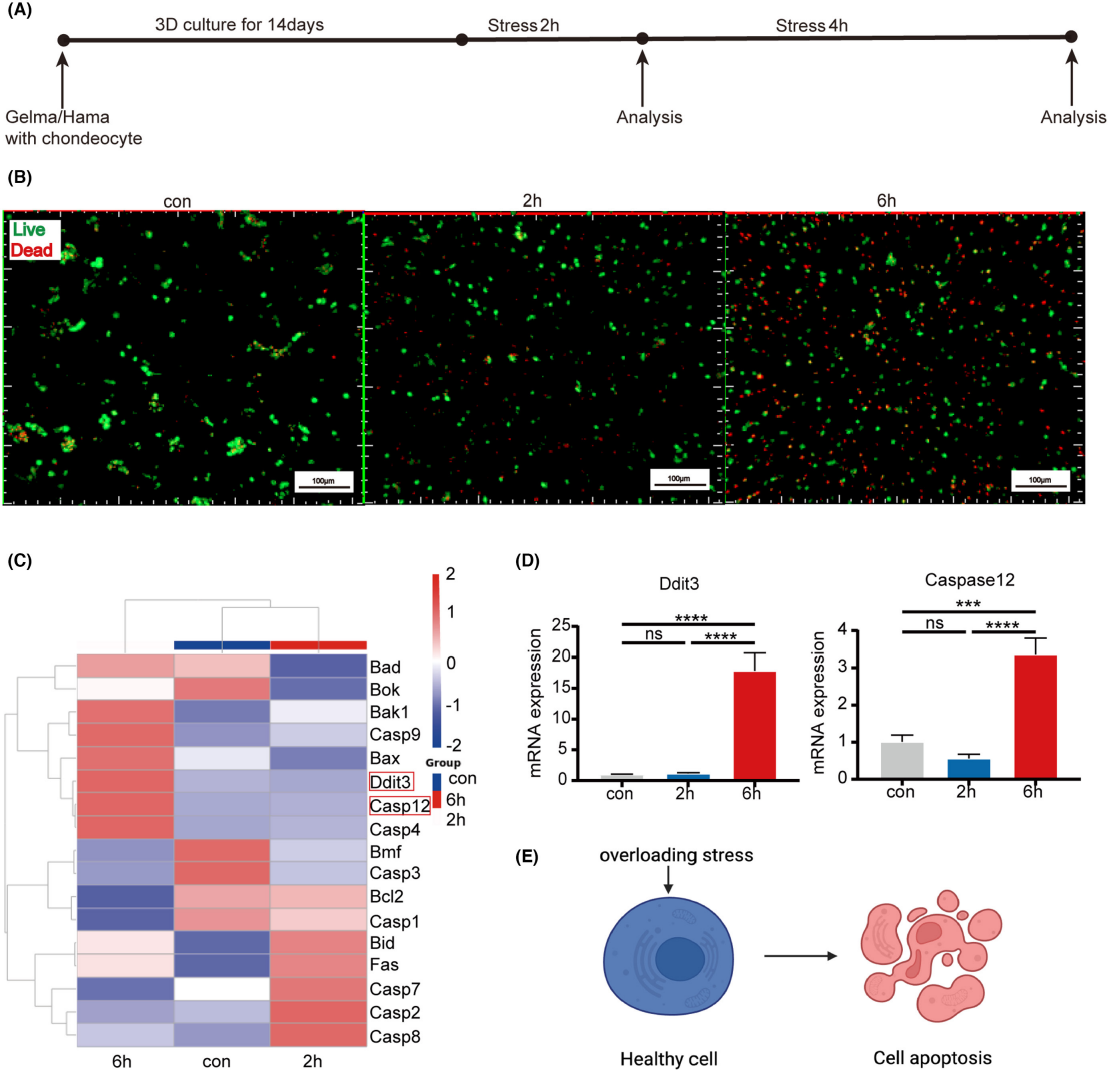
Overloading pressure‐induced apoptosis in chondrocytes. (A) Mechanical loading procedure for cell‐laden hydrogel constructs. (B) Representative images of live‐dead staining for chondrocytes cultured in GelMA/HAMA hydrogel after 0, 2, and 6 h of compression green fluorescence indicates live cells and red fluorescence indicates dead cells. Scare bars: 200 μm. (C) Heatmaps depicting the differential expression of apoptosis‐related genes in chondrocytes after 0, 2, and 6 h of compression. (D) The relative expression of Ddit3 and Caspase12 in chondrocytes after 0, 2, and 6 h of compression. Data were presented as the mean ± SD (*n* = 3). (E) A model demonstrating pressure‐induced apoptosis. The results were analysed by one‐way anova followed by Tukey's test ***p < 0.001, and ****p < 0.0001. ns, no significance.

Next, we utilized a pressure system to evaluate the impact of excessive mechanical loading on chondrocyte apoptosis. Initially, we explored the effects of compression duration on chondrocytes (Figure 1A; Figure S3A,B). Live/dead cell staining images revealed a noticeable increase in deceased chondrocytes after 6 h of compression (Figure 1B). RNASeq analysis conducted on chondrocytes post 0, 2, and 6 h of compression demonstrated that mechanical pressure upregulated the expression of apoptosis‐related genes. Notably, there was a significant increase in the expression of ERS‐related apoptotic genes, particularly after 6 h of compression (Figure 1C). qRT‐PCR results confirmed a substantial upregulation in the expression of ERS‐related apoptotic genes, including ddit3 and caspase12, after 6 h of compression (Figure 1D). The findings indicate that excessive pressure can induce chondrocyte apoptosis (Figure 1E).

### 3.4 Overloading pressure induces ERS in chondrocytes

**FIGURE 2:**
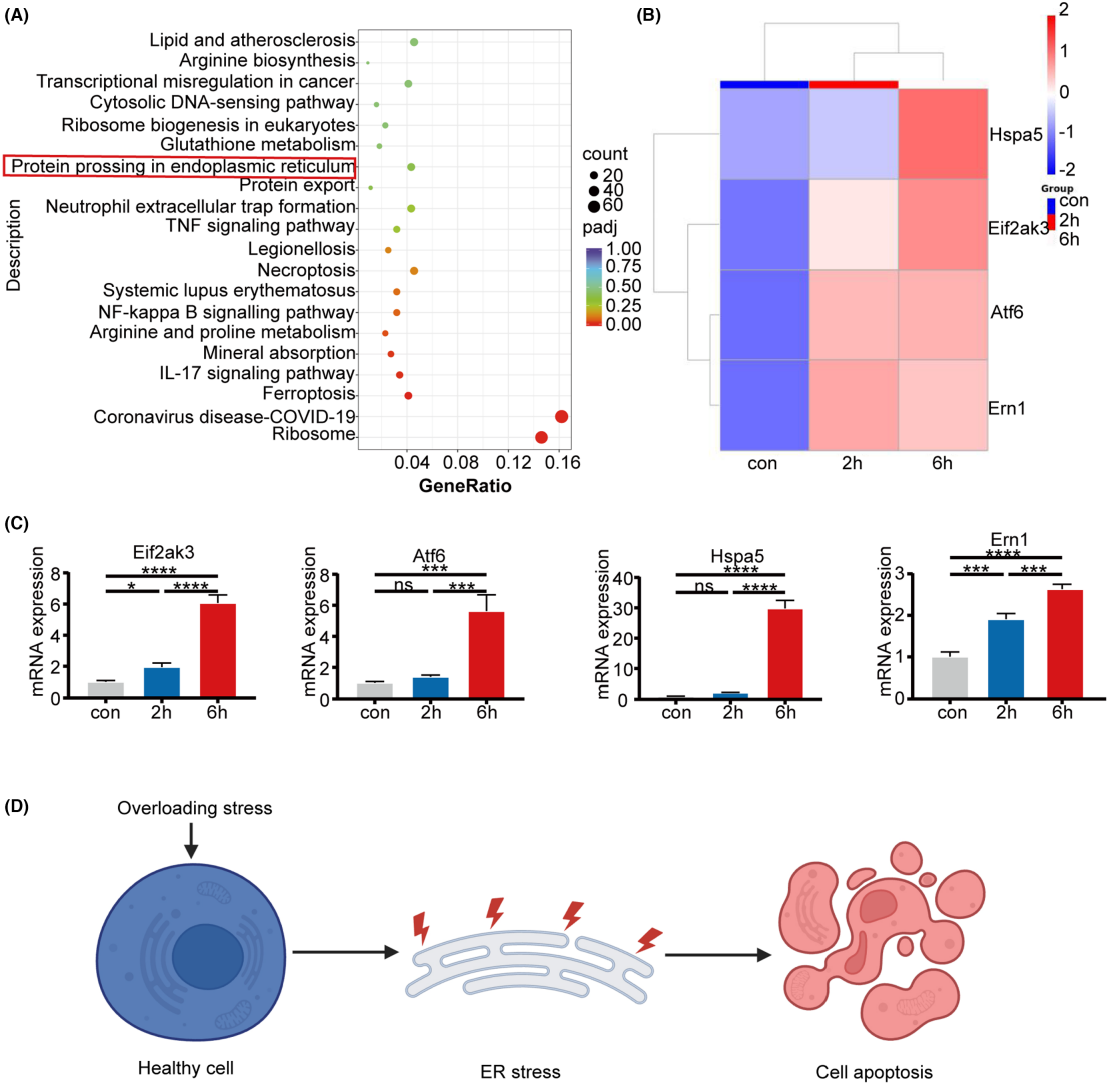
Overloading pressure leads to ERS in chondrocytes. (A) Kyoto Encyclopedia of Genes and Genomes (KEGG) pathway enrichment analysis. (B) Heatmaps depict the differential expression of ERS‐related genes in chondrocytes after 0, 2, and 6 h of stress. (C) Relative expression levels of ERN1, PERK, HSPA5, and ATF6 in chondrocytes following 0, 2, and 6 h of compression. (D) A model illustrating pressure‐induced apoptosis through ERS. Statistical analysis using one‐way anova followed by Tukey's test. Data are presented as mean ± SD (*n* = 3). *p < 0.05, ***p < 0.001, and ****p < 0.0001. ns, no significance.

Kyoto Encyclopedia of Genes and Genomes (KEGG) pathway analysis indicated a correlation between ERS‐related proteins and increased pressure (Figure 2A). Moreover, the upregulation of ERS markers ERN1, PERK, HSPA5, and ATF6 was observed following compression (Figure 2B). The RNAseq findings were validated through qRT‐PCR (Figure 2C,D).

### 3.5 Overloading pressure leads to an upregulation of Piezo1 expression on the chondrocyte membrane

**FIGURE 3:**
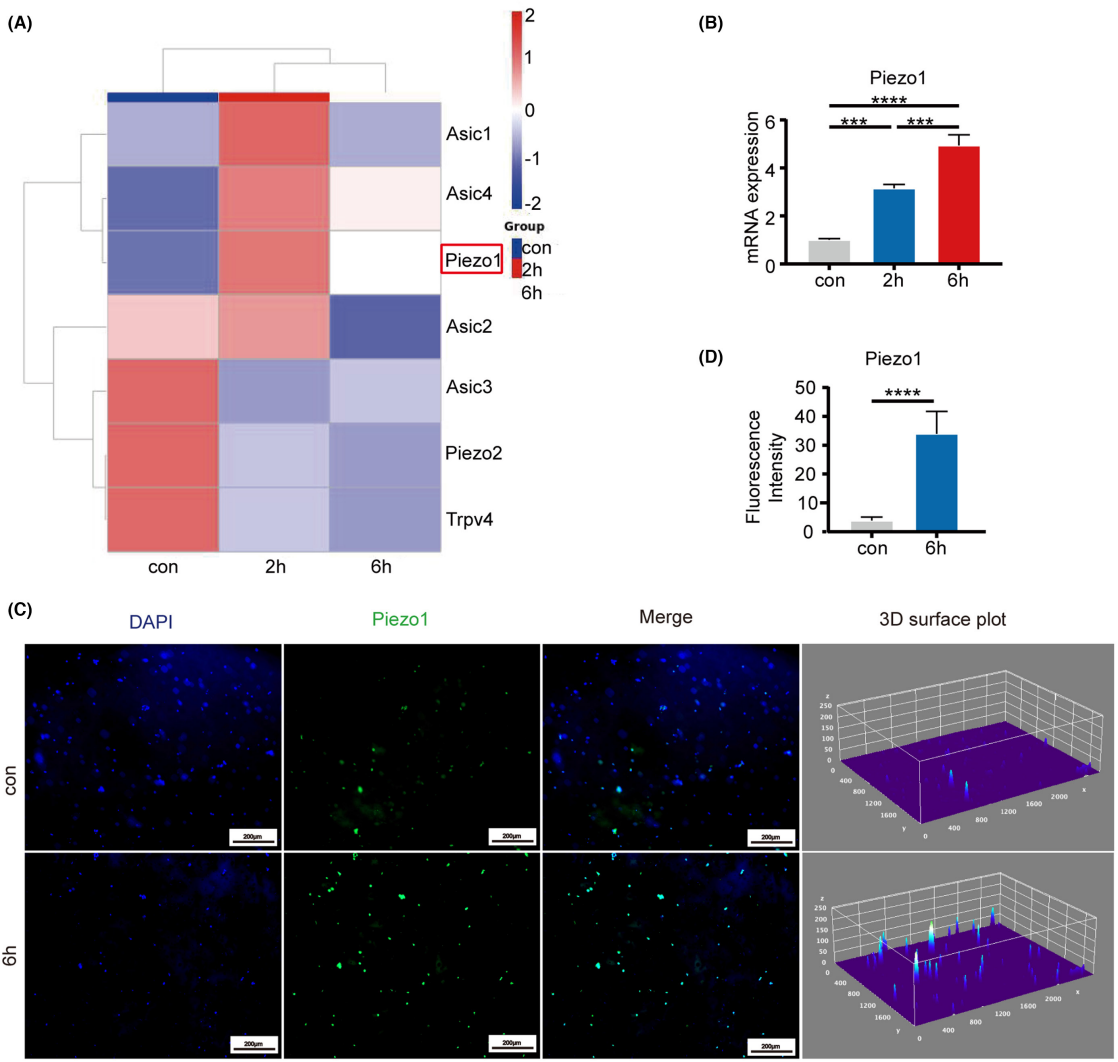
Overloading pressure results an elevation in the expression of Piezo1 in chondrocytes. (A) Heatmaps depict the differential expression of MA channel genes in chondrocytes after 0, 2, and 6 h of stress. (B) Relative expression levels of Piezo1 in chondrocytes after 0, 2, and 6 h of compression. (C) Representative images and a 3D surface plot of Piezo1 antibody IF staining in chondrocytes exposed to overloading pressure for 0 or 6 h. (D) Quantification of (C). Statistical analysis using one‐way anova followed by Tukey's test. Data are presented as mean ± SD (*n* = 3). ***p < 0.001 and ****p < 0.0001. ns, no significance.

To explore the correlation between MA ion channels on chondrocyte membranes and excessive pressure, we conducted gene sequencing. After screening for MA channel‐related genes, the results revealed a marked increase in Piezo1 expression post pressure loading (Figure S4A,B; Figure 3A). Consistency between the qRT‐PCR and IF staining results and the RNA sequencing findings was observed (Figure 3B–D). Meanwhile, qRT‐PCR results indicate that GsMTx4 exerts inhibitory effects on the upregulation of Piezo1 expression induced overloading load (Figure S4C).

### 3.6 Piezo1 mediates the influx of extracellular calcium ions into the cell, triggering ERS and apoptosis in chondrocytes

**FIGURE 4:**
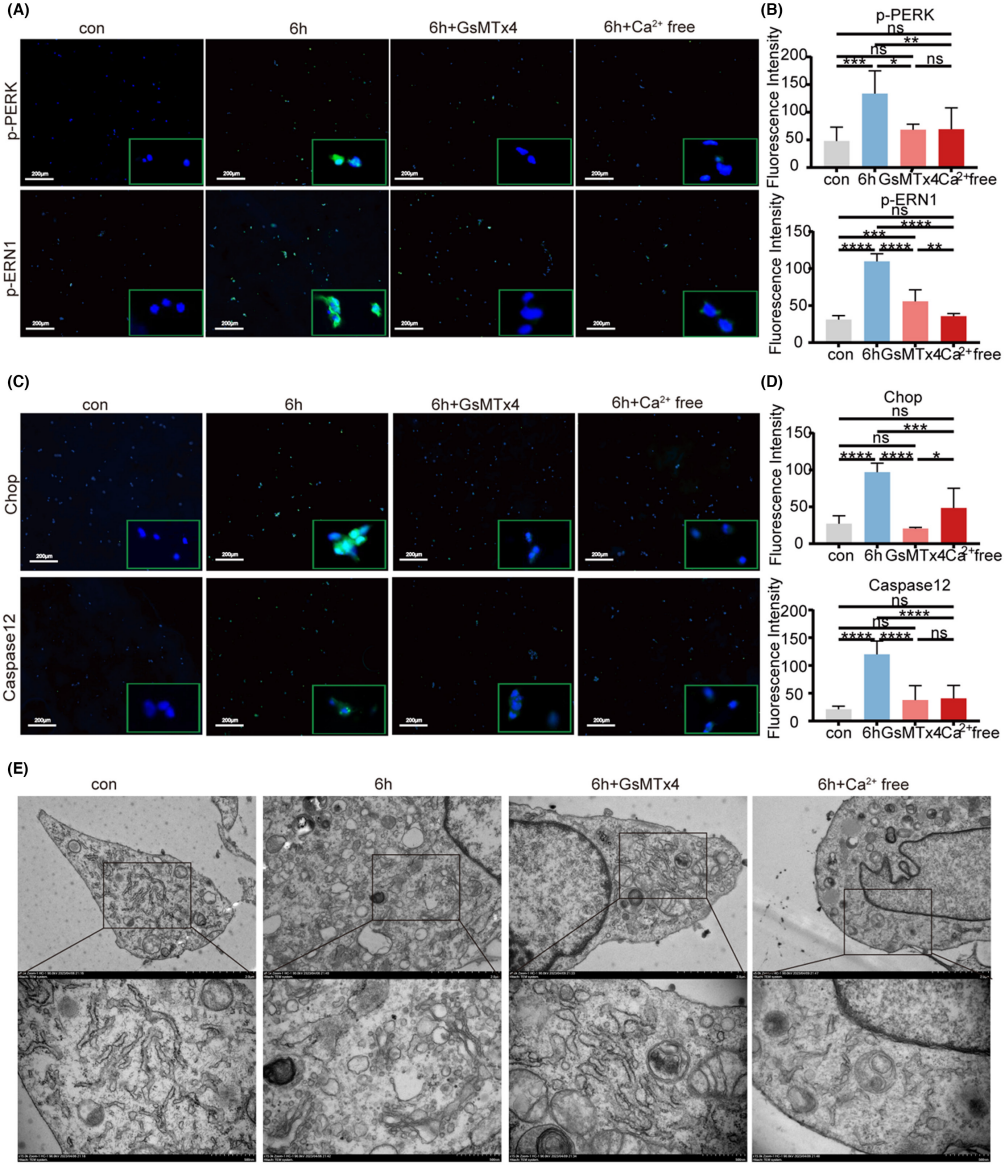
Piezo1 mediates the influx of extracellular calcium ions into the cell, triggering ERS and apoptosis in chondrocytes. (A) Chondrocytes were exposed to overloading pressure for 6 h and treated with GsMTx4 or medium without calcium (Ca^2+^free). Representative images of IF staining of p‐PERK and p‐ERN1 in chondrocytes. Scale bars: 200 μm. (B) Quantification of (A) (*n* = 6). (C) Representative images of IF staining of Chop and Caspase12 in chondrocytes. Scale bars: 200 μm. (D) Quantification of (C) (*n* = 6). (E) Chondrocytes were subjected to injurious mechanical pressure for 0, 6 h and treated with GsMTx4 or medium without calcium (Ca^2+^free). TEM images of chondrocytes with 6000× and 15,000× are shown. Statistical analysis using one‐way anova followed by Tukey's test. All data are expressed as the mean ± SEM *p < 0.05, **p < 0.01, ***p < 0.001, and ****p < 0.0001. ns, no significance.

To explore the correlation between Piezo1 and ERS, as well as apoptosis in chondrocytes, we utilized GsMTx4 and a calcium‐free culture medium. qRT‐PCR and IF staining results indicated that GsMTx4 suppresses the expression of ERS and apoptosis‐related genes and proteins in chondrocytes. Moreover, in the absence of GsMTx4, the elimination of extracellular calcium ions similarly decreased endoplasmic reticulum stress and apoptosis in chondrocytes (Figure 4A–D; Figure S5A,B). TEM analysis revealed that untreated chondrocytes exhibited a continuous and dense endoplasmic reticulum with fewer cytoplasmic vesicles. Overloading pressure caused the endoplasmic reticulum in chondrocytes to expand, resulting in the disappearance of rough endoplasmic reticulum, clustering of swollen mitochondria, the presence of numerous round vacuoles in the cytoplasm, and notable evidence of apoptosis. Treatment with GsMTx4 and a calcium‐free culture medium mitigated the extent of endoplasmic reticulum expansion, reduced vesicles, and alleviated the degree of apoptosis (Figure 4E).

### 3.7 In vivo, overloading pressure induces ERS and apoptosis in chondrocytes via the Piezo1 ion channel

**FIGURE 5:**
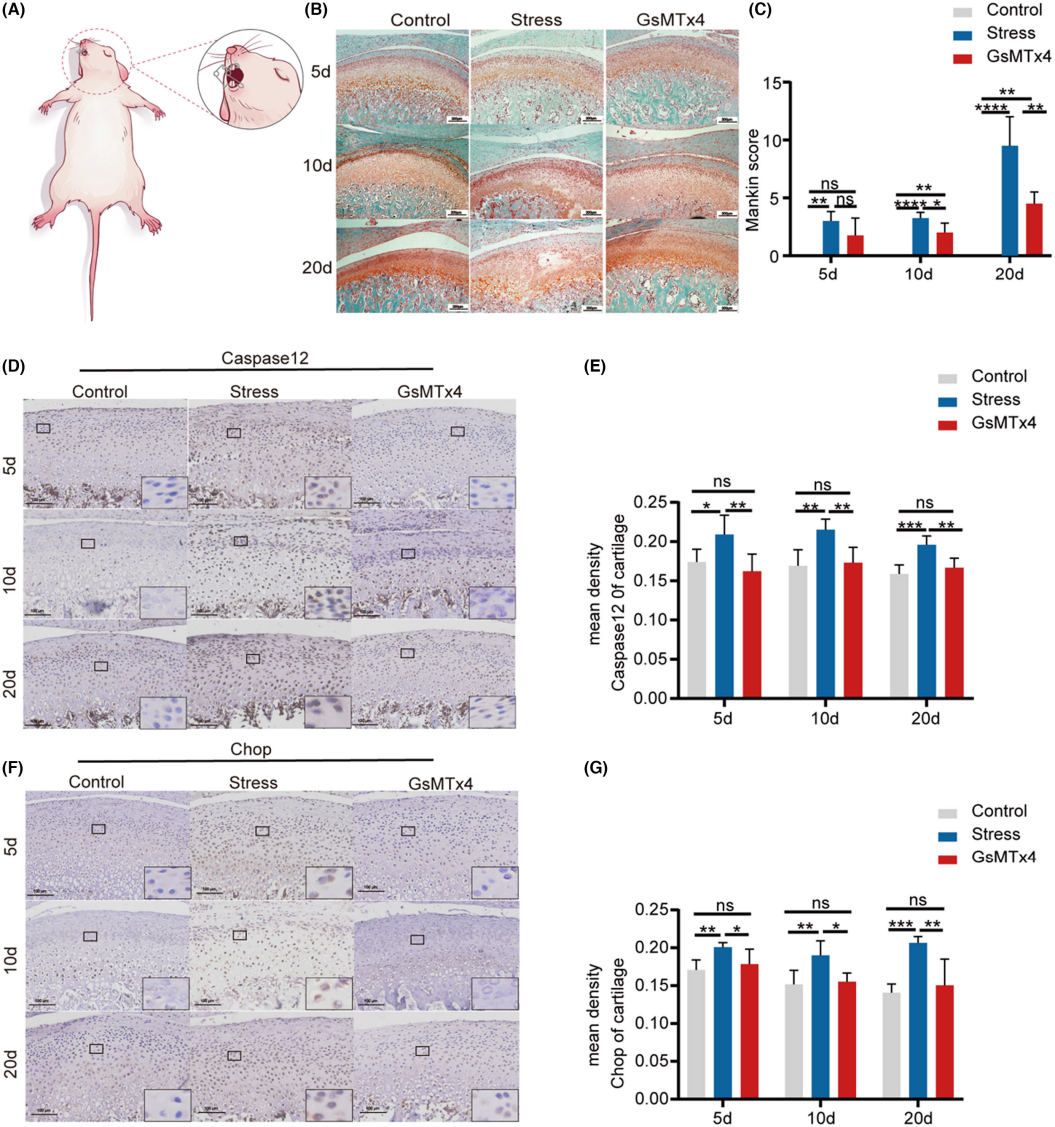
Overloading pressure induces ERS and apoptosis in chondrocytes through the piezo1 ion channel. (A) The model of forced‐mouth opening. (B) SO&FG staining. (C) The Mankin score of cartilage layers. The data are expressed as the mean ± SEM (*n* = 3). (D–G) Caspase12 and chop expression evaluated by immunohistochemistry. All data are expressed as the mean ± SEM (*n* = 6). The results were analysed by one‐way anova followed by Tukey's test. *p < 0.05, **p < 0.01, ***p < 0.001, and ****p < 0.0001. Control: negative control group. Stress: forced mouth‐opening model group. GsMTx4: treatment group.

To investigate Piezo1's role in chondrocytes, we induced a forced‐mouth opening model in Sprague–Dawley rats (Figure 5A). The forced mouth‐opening group showed evident cartilage thinning, while the GsMTx4 treatment group exhibited milder cartilage alterations (Figure S6A). Safranin O‐fast green staining revealed significant proteoglycan loss, reduced cartilage staining area, and disrupted cartilage structure in the forced‐mouth opening model group. Conversely, the GsMTx4 treatment group exhibited limited proteoglycan loss (Figure 5B). Mankin scores in the GsMTx4 treatment group were lower than those in the forced mouth‐opening model group, with no cartilage damage observed in the control group (Figure 5C).

Besides TMJ cartilage investigation, we assessed changes in TMJ subchondral bone microstructure using Micro‐CT. In the 20‐day group, sagittal sections of subchondral bone in the forced‐mouth opening model group showed progressive destruction and significant trabecular loss, particularly in the posterior region. These changes were less pronounced in the GsMTx4 treatment group compared to the model group (Figure S6B). In the 20‐day group, bone volume fraction (Bv/Tv), trabecular thickness (Tb. Th), and trabecular number (Tb. N) were lower in the model group than the control group. Conversely, the GsMTx4 treatment group exhibited a significant increase in subchondral bone BV/TV, Tb. Th, and Tb. N compared to the model group. However, trabecular separation (Tb. Sp) was higher in the model group than the control group, with the GsMTx4 treatment group showing a significantly lower value than the model group (Figure S6C).

To verify the transmission of mechanical pressure through the Piezo1 ion channel in the forced mouth‐opening model, we conducted immunohistochemical staining to assess Piezo1 expression. The forced mouth‐opening model group demonstrated a substantial increase in Piezo1 expression in the proliferative cartilage zone compared to the control group. The GsMTx4 group exhibited reduced Piezo1 expression compared to the forced mouth‐opening group (Figure S7A,B).

Subsequently, to evaluate ERS and apoptosis in chondrocytes, immunohistochemical staining revealed a significant upregulation of p‐PERK, p‐ERN1, Caspase‐12, and Ddit3 expression in the cartilage layer of the model group. In contrast, the GsMTx4 treatment group exhibited a significant downregulation of these protein expressions (Figure 5D–G; Figure S7C–F).

## 4 DISCUSSION

**FIGURE 6:**
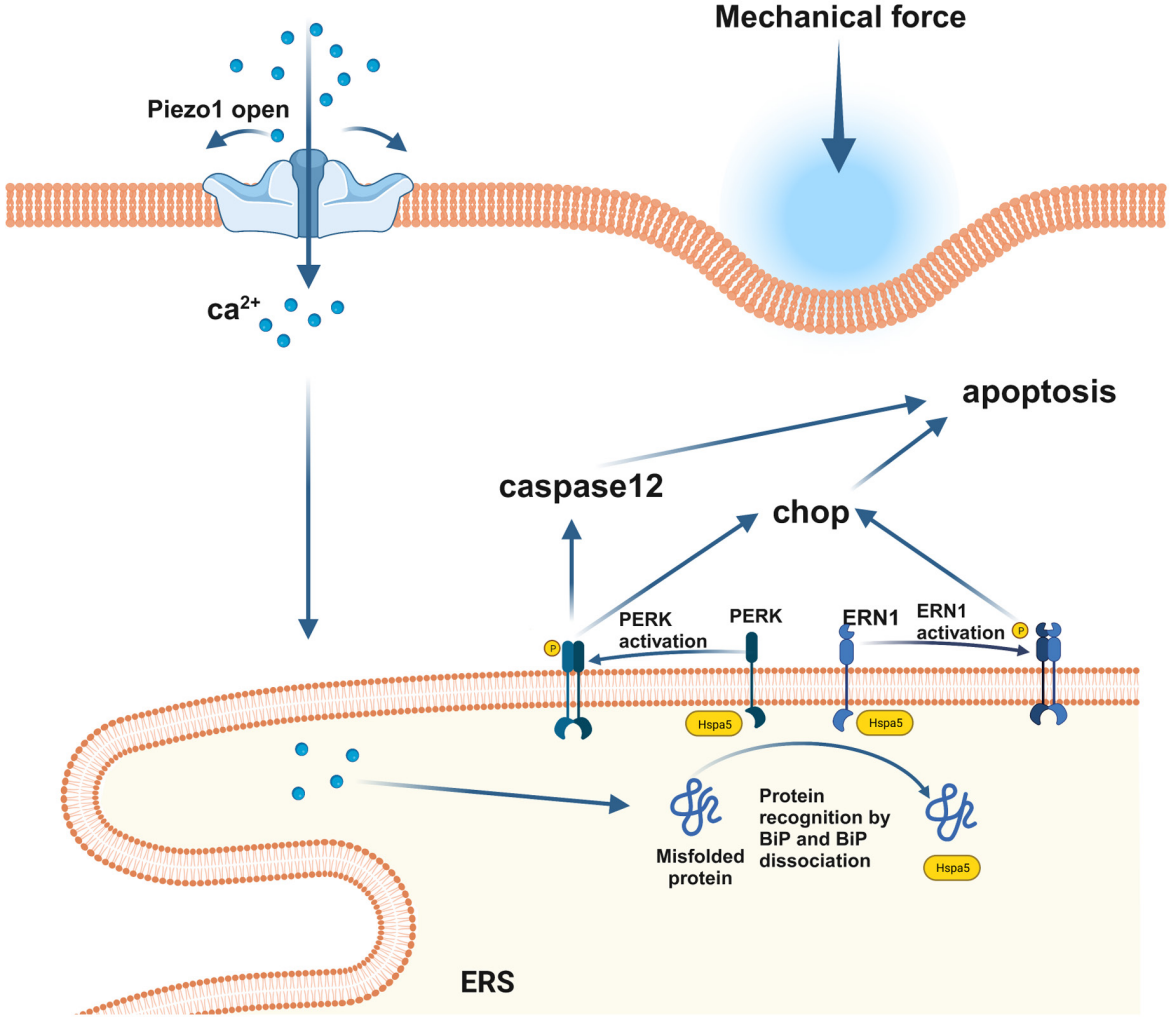
A working model in which overloading stress induces ERS in chondrocytes, leading to cellular apoptosis.

In this study, we showed that overloading pressure in TMJ chondrocytes induces calcium influx through the Piezo1 ion channel, subsequently activating the p‐PERK and p‐ERN1‐mediated ERS apoptosis pathway (Figure 6). The findings shed light on a partial mechanistic role of Piezo1 in chondrocyte apoptosis and offer insights for further exploration into the pathogenesis and treatment strategies of osteoarthritis.

TMJOA is a common degenerative joint disorder characterized by degenerative changes in the articular cartilage. The primary cause of these changes is likely an increase in chondrocyte apoptosis.^36^ Therefore, exploring the mechanisms of chondrocyte apoptosis in joint cartilage is significant for targeted therapy of TMJ osteoarthritis. The aetiology of TMJ condylar cartilage degeneration is multifaceted, involving malocclusion, trauma, excessive loading, individual development, genetic factors, and systemic diseases like rheumatoid arthritis.^37^ However overloading stress stands out as a significant etiological factor in TMJ osteoarthritis, contributing to the progressive degeneration of temporomandibular condylar cartilage and hindering chondrocyte self‐repair.^31^ Studies have highlighted a notable correlation between mouth breathing in adolescents and condylar morphology. In cases of partial condylar resorption, correction of mouth breathing halts the resorption process.^38^ In this study, reconstructing the condyle after addressing mouth breathing implies a strong link between excessive load and articular cartilage degeneration. However, there is limited literature on the specific mechanisms and occurrence of chondrocyte apoptosis induced by excessive load.

Simulating the in vivo environment and force conditions of chondrocytes in vitro is crucial for studying the impact of excessive load. Light‐crosslinked hydrogels, such as GelMA and HAMA, are widely employed as tissue engineering scaffolds.^39^, ^40^ In this study, we employed these hydrogels to mimic in vivo conditions. Safranin O staining revealed significant ECM secretion by chondrocytes after three‐dimensional cultivation, confirming successful simulation of the in vivo environment. Selecting an appropriate pressure application method is essential for mimicking force conditions in vitro. We used the FX‐5000C™ FLEXCELL® Compression Plus™ System to apply excessive load to in vitro cultured chondrocytes.^41^ Consistent with clinical observations, under overloading pressure, chondrocyte death increased, accompanied by upregulation of apoptosis‐related genes Ddit3 and Caspase‐12. This indicates the ability of excessive load to activate apoptosis in chondrocytes.

Previous research indicates that cell apoptosis is a genetically controlled process. Apoptosis in eukaryotic cells is mainly linked to death receptor‐mediated extrinsic pathways, mitochondrial‐mediated intrinsic pathways, or endoplasmic reticulum stress‐mediated pathways.^42^ In our experiments, applying excessive pressure to chondrocytes resulted in elevated endoplasmic reticulum protein synthesis, as indicated by KEGG pathway analysis. Physiological pressure on cells leads to elevated calcium ions, causing ER calcium overload. This results in the accumulation of misfolded proteins, increased co‐expression of the molecular chaperone HSPA5, an upregulation in the expression of the molecular chaperone HSPA5, and activation of ER‐localized sensors like ERN1, PERK, and ATF6. These processes collectively adjust endoplasmic reticulum stress in response to elevated calcium ion levels in the cell.^43^, ^44^, ^45^ However, under abnormal fluid shear stress, normal cellular homeostasis cannot be restored. The ERS apoptosis pathway in chondrocytes is activated through p‐PERK and p‐ERN1 mediation.^46^ In line with our experimental results, overloading pressure upregulates the expression of ERS‐related genes, including ERN1, PERK, ATF6, and HSPA5 and promotes the phosphorylation of ERN1 and PERK. This suggests that overloading pressure can induce chondrocyte apoptosis through the ERS‐apoptosis pathway.

The accumulation of calcium ions in the ER induces ERS in chondrocytes, while mechanically activated ion channels facilitate extracellular calcium influx. Recent research identifies four primary mechanosensitive ion channels on the chondrocyte membrane: Piezo1, Piezo2, Trpv4, and ASICs.^47^, ^48^ Trpv4, among these channels, responds to various stimuli including mechanical, thermal, and chemical cues, influencing chondrocyte metabolism and matrix synthesis through Ca^2+^ influx and affecting other physiological processes. Studies suggest that TRPV4 ion channel mediates synthetic metabolic responses to physiological mechanical signals in chondrocytes.^6^ However, in vitro investigations also indicate that TRPV4 activation may trigger catabolic metabolism and pro‐inflammatory reactions in chondrocytes, potentially leading to chondrocyte necroptosis.^49^ ASICs, another group of ion channels sensitive to acidic stimuli, exhibit responsiveness to mechanical stimulation in chondrocytes as well. ASICs activity in chondrocytes could participate in regulating cellular function and metabolism in acidic environments. Piezo1 and Piezo2, while structurally similar, serve distinct physiological roles. Piezo2 is mainly found in sensory neurons, detecting external mechanical stimuli, whereas Piezo1 primarily senses harmful mechanical stimuli outside the cell.^50^, ^51^, ^52^ Prior studies have identified Piezo1 as a pivotal mechanically activated ion channel that regulates intracellular calcium homeostasis. Upon mechanical stimulation, Piezo1 channels open, enabling calcium entry into cells.^50^, ^51^, ^52^ However, the specific mechanism by which Piezo1 mediates chondrocyte apoptosis under excessive load remains unexplored. Our experiments demonstrate that excessive pressure upregulates Piezo1 expression in chondrocytes. Elevated or hyperactivated Piezo1 expression may lead to uncontrolled calcium‐dependent apoptosis.^14^, ^53^ In our experiments, activated Piezo1 channels concurrently triggered p‐PERK and p‐ERN1‐mediated ERS apoptosis. Inhibiting Piezo1 activity or reducing intracellular calcium ions mitigated these changes. This underscores the crucial role of the Piezo1 ion channel in chondrocyte ERS apoptosis induced by excessive load.

We expanded our previous observations to the forced‐mouth opening model in SD rats, a validated model known to induce TMJ cartilage degeneration and subchondral bone changes.^54^, ^55^, ^56^ TMJ cartilage degeneration is one of the key pathological manifestations of TMJOA. In our experiments, the forced‐mouth opening model increased Piezo1 expression in the proliferative layer of TMJ cartilage, concurrently activating phosphorylation of ERS‐associated proteins linked to chondrocyte apoptosis. Safranin O‐fast green staining and micro‐CT results further confirmed that overloading pressure resulted in TMJ cartilage degradation and subchondral bone loss. The GsMTx4 treatment group exhibited less severe cartilage degeneration and subchondral bone loss compared to the model group, indicating a potential therapeutic effect of GsMTx4 on overloading pressure‐induced TMJOA.

This study has certain limitations. It primarily focuses on the impact of excessive pressure on TMJ chondrocytes, highlighting the interconnected nature of the joint environment. Further in‐depth investigations into synovium, joint disc, and other related tissues are warranted. Additionally, the forced‐mouth opening model, as an artificial stimulus, requires further refinement to align more closely with the natural progression of TMJ osteoarthritis. Future research should adopt a more comprehensive approach, taking into account various factors, to enhance our understanding of the pathogenesis of TMJ osteoarthritis.

In conclusion, our study establishes the critical role of Piezo1 in TMJ osteoarthritis. The partial inhibitory effect of GsMTx4 offers a new perspective for treating TMJ osteoarthritis, emphasizing the innovative and novel contributions of this research to the field of osteoarthritis.

## AUTHOR CONTRIBUTIONS

**Xiaohui Wang:** Data curation (lead); formal analysis (lead); methodology (lead); writing – original draft (lead); writing – review and editing (lead). **Junli Tao:** Data curation (lead); formal analysis (lead); methodology (lead); writing – original draft (lead); writing – review and editing (equal). **Jianping Zhou:** Formal analysis (equal); funding acquisition (equal). **Yi Shu:** Resources (equal); validation (equal); writing – review and editing (equal). **Jie Xu:** Funding acquisition (equal); resources (equal); writing – original draft (equal); writing – review and editing (equal).

## CONFLICT OF INTEREST STATEMENT

The authors declare that they have no competing interests.

## Supporting information


Figure S1:

Figure S2:

Figure S3:

Figure S4:

Figure S5:

Figure S6:

Figure S7:

## Data Availability

The data that support the findings of this study are available on request from the corresponding author.
