# Supplementary material for: Excessive load promotes temporomandibular joint chondrocyte apoptosis via Piezo1/endoplasmic reticulum stress pathway
Source: J Cell Mol Med. 2024 Jun 6;28(11):e18472. doi: 10.1111/jcmm.18472 (PMC11154833; doi:10.1111/jcmm.18472)
Supplement: Supplementary file 1 — Figure S1: [file JCMM-28-e18472-s002.docx]

Supplementary Materials:


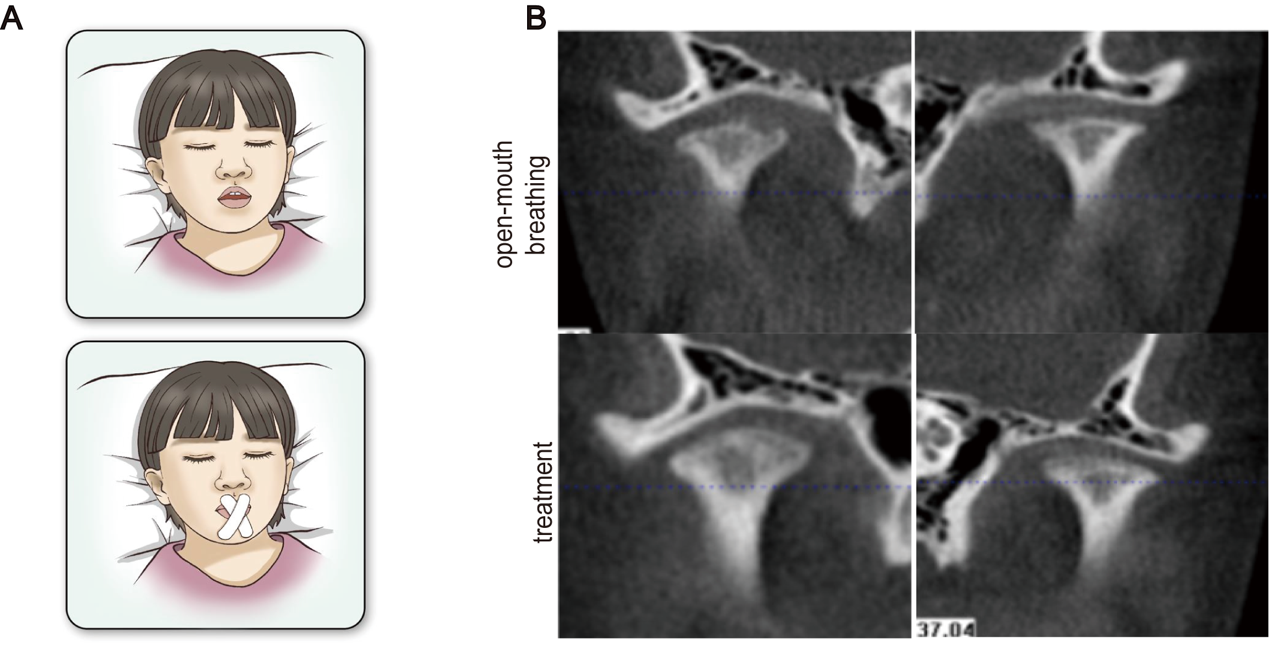


**Figure S1** Mouth breathing causes changes in the condyle (A) The model of mouth breathing and its treatment. (B) Representative morphological features of the condyle in open-mouth breathing, and treatment group.
