# Supplementary material for: Excessive load promotes temporomandibular joint chondrocyte apoptosis via Piezo1/endoplasmic reticulum stress pathway
Source: J Cell Mol Med. 2024 Jun 6;28(11):e18472. doi: 10.1111/jcmm.18472 (PMC11154833; doi:10.1111/jcmm.18472)
Supplement: Supplementary file 2 — Figure S2: [file JCMM-28-e18472-s004.docx]

Supplementary Materials:


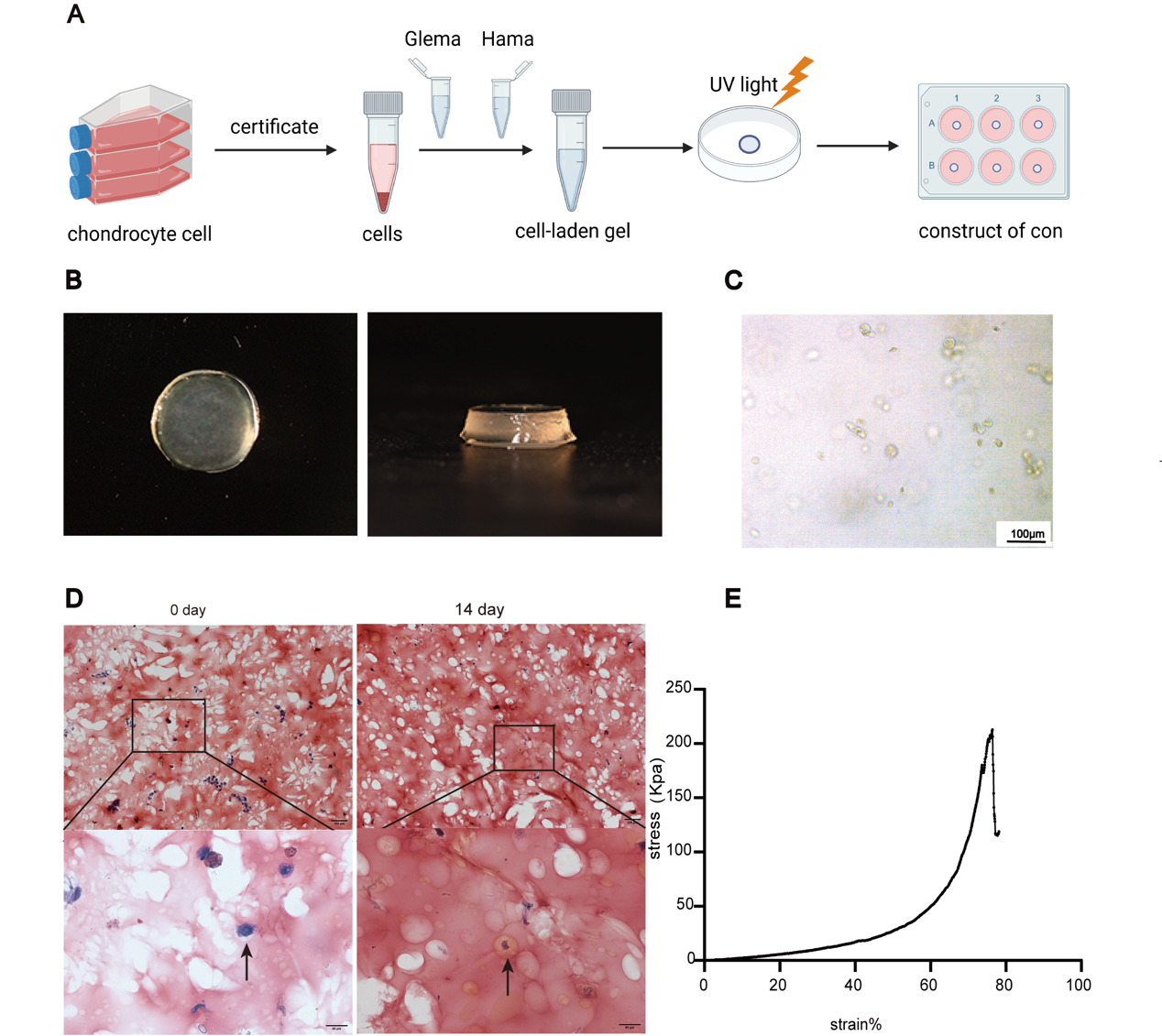


**Figure S2**Three-dimensional in vitro culture of chondrocytes (A) The schematic diagram of the production processes of cell-laden hydrogel constructs. (B) The shape of cell-laden hydrogel constructs. (C)Chondrocytes cultured in 3D maintain their round morphology. (D) Representative images of safranin-O staining of chondrogenic induction culture. Scale bars: above, 100 μm; below, 50 μm. (E) the stress-strain curve of cell-laden hydrogel constructs.
