# Supplementary material for: Excessive load promotes temporomandibular joint chondrocyte apoptosis via Piezo1/endoplasmic reticulum stress pathway
Source: J Cell Mol Med. 2024 Jun 6;28(11):e18472. doi: 10.1111/jcmm.18472 (PMC11154833; doi:10.1111/jcmm.18472)
Supplement: Supplementary file 3 — Figure S3: [file JCMM-28-e18472-s006.docx]

Supplementary Materials:


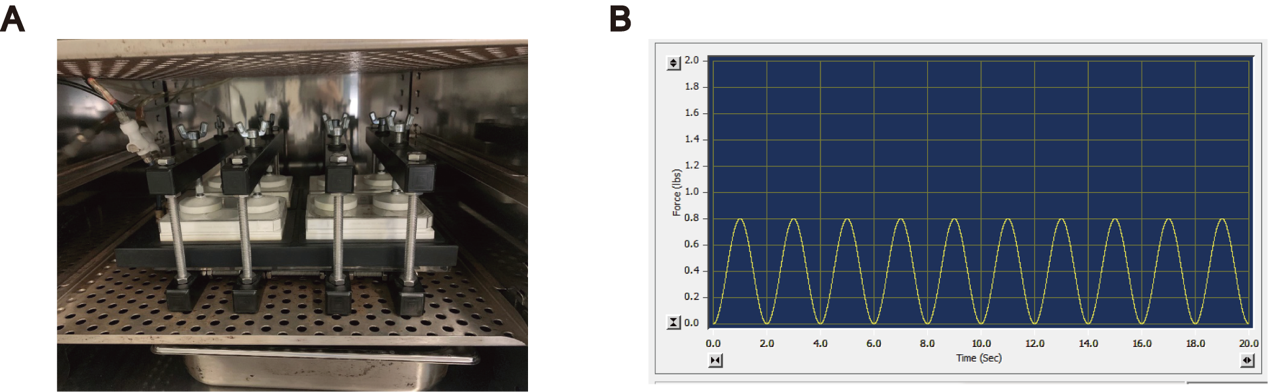


**Figure S3 (**A) The FX-5000C™ FLEXCELL® Compression Plus™ System. (B) Mechanical pressure protocol.
