# Supplementary material for: Excessive load promotes temporomandibular joint chondrocyte apoptosis via Piezo1/endoplasmic reticulum stress pathway
Source: J Cell Mol Med. 2024 Jun 6;28(11):e18472. doi: 10.1111/jcmm.18472 (PMC11154833; doi:10.1111/jcmm.18472)
Supplement: Supplementary file 4 — Figure S4: [file JCMM-28-e18472-s003.docx]

Supplementary Materials:


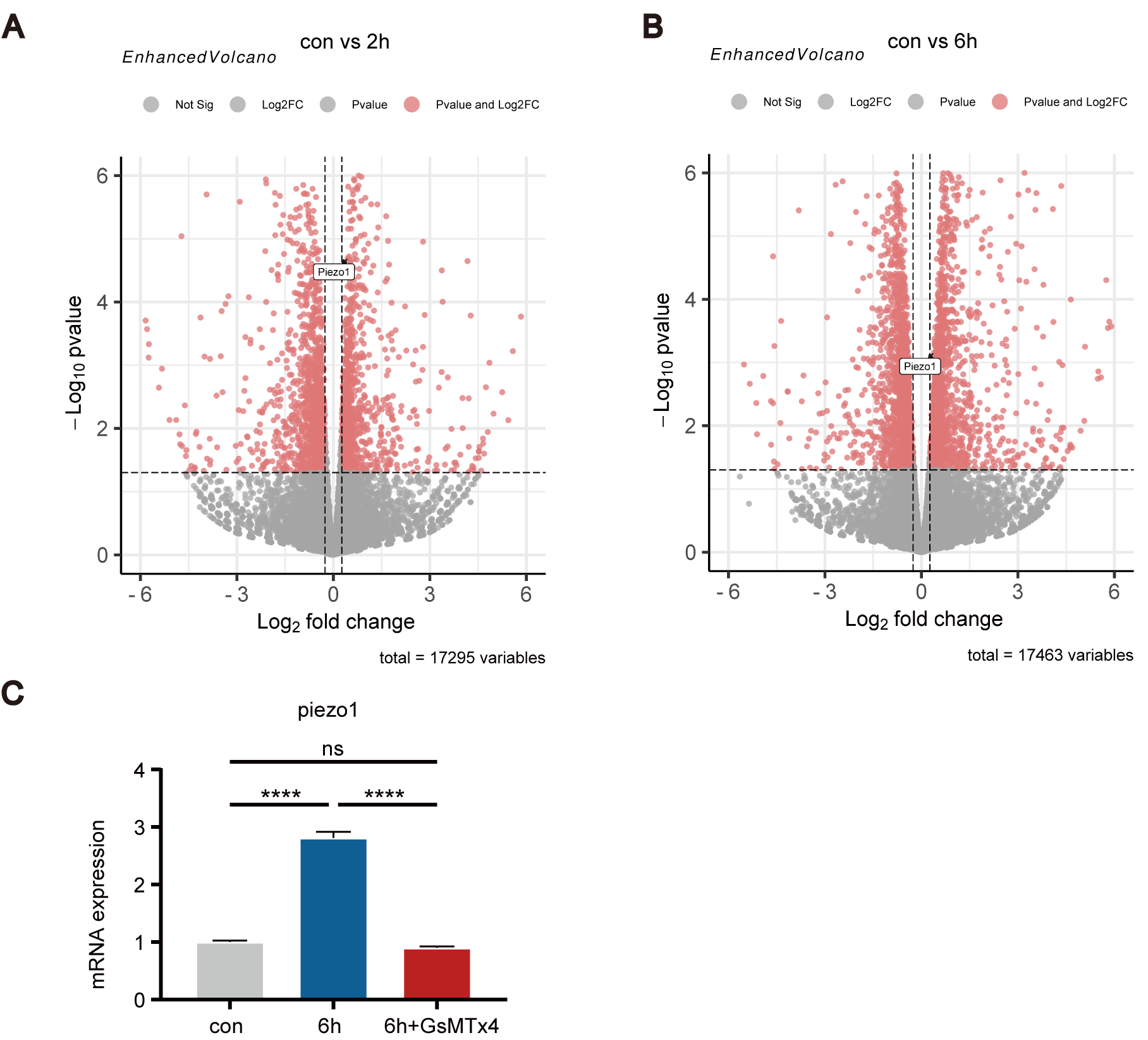


**Figure S4** (A) The expression of Piezo1 exhibits a significant difference between the control group and the pressurized group after two hours. (B) The expression of Piezo1 exhibits a significant difference between the control group and the pressurized group after six hours. (C)The relative expression of Piezo1 in chondrocytes after 0, and 6 hours of compression with or without GSMT×4.
