# Supplementary material for: Excessive load promotes temporomandibular joint chondrocyte apoptosis via Piezo1/endoplasmic reticulum stress pathway
Source: J Cell Mol Med. 2024 Jun 6;28(11):e18472. doi: 10.1111/jcmm.18472 (PMC11154833; doi:10.1111/jcmm.18472)
Supplement: Supplementary file 5 — Figure S5: [file JCMM-28-e18472-s001.docx]

Supplementary Materials:


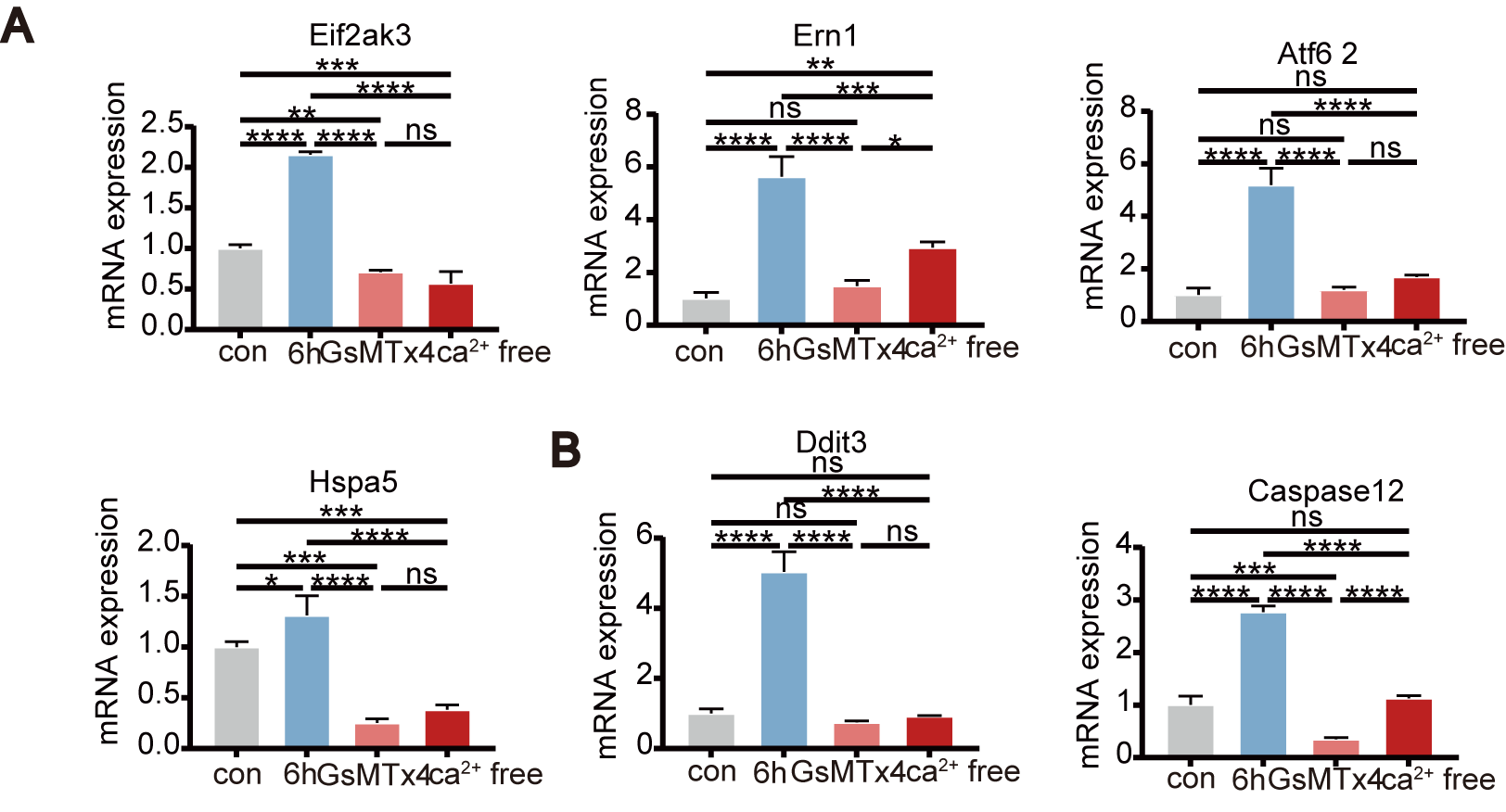


**Figure S5** (A) Chondrocytes were exposed to Overloading pressure for 6 h and treated with GSMT×4 or medium without calcium (Ca^2+^-free). qRT-PCR analyses for the mRNA expression of Hspa5, Eif2ak3, Ern1 and Atf6 (n=3). (B) qRT-PCR analyses for the mRNA expression of Chop and Caspase12 (n=3). Statistical analysis using one-way ANOVA followed by Tukey’s test. Data are presented as mean ± SD (n=3). *p< 0.05, **p< 0.01, ***p< 0.001, and ****p< 0.0001, ns, no significance.
