# Supplementary material for: Excessive load promotes temporomandibular joint chondrocyte apoptosis via Piezo1/endoplasmic reticulum stress pathway
Source: J Cell Mol Med. 2024 Jun 6;28(11):e18472. doi: 10.1111/jcmm.18472 (PMC11154833; doi:10.1111/jcmm.18472)
Supplement: Supplementary file 6 — Figure S6: [file JCMM-28-e18472-s005.docx]

Supplementary Materials:


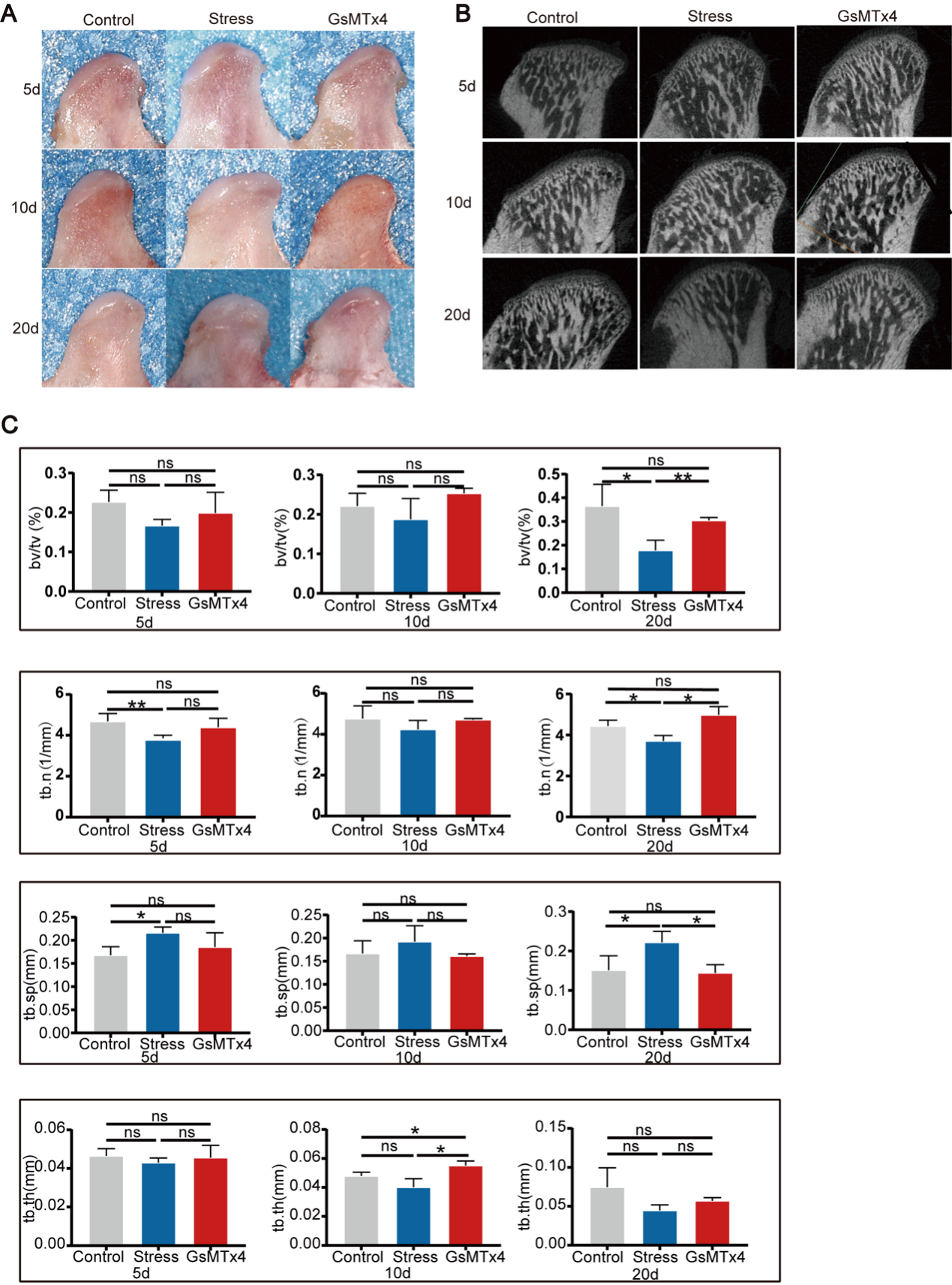


**Figure S6** (A) Representative morphological features of the condyle in control, model, and treatment group. (B) The middle picture of TMJ by micro-CT Scanning. (C) Quantitative analysis of the structural parameters of subchondral bone by micro‐CT. The data are expressed as the mean±SEM (n=6). The results were analyzed by one-way ANOVA followed by Tukey’s test. ∗P<0.05, ∗∗P<0.01, and∗∗∗P<0.001. Control: negative control group. Stress: forced mouth-opening model group. GsMTx4: treatment group.
