# Supplementary material for: Excessive load promotes temporomandibular joint chondrocyte apoptosis via Piezo1/endoplasmic reticulum stress pathway
Source: J Cell Mol Med. 2024 Jun 6;28(11):e18472. doi: 10.1111/jcmm.18472 (PMC11154833; doi:10.1111/jcmm.18472)
Supplement: Supplementary file 7 — Figure S7: [file JCMM-28-e18472-s007.docx]

Supplementary Materials:

**
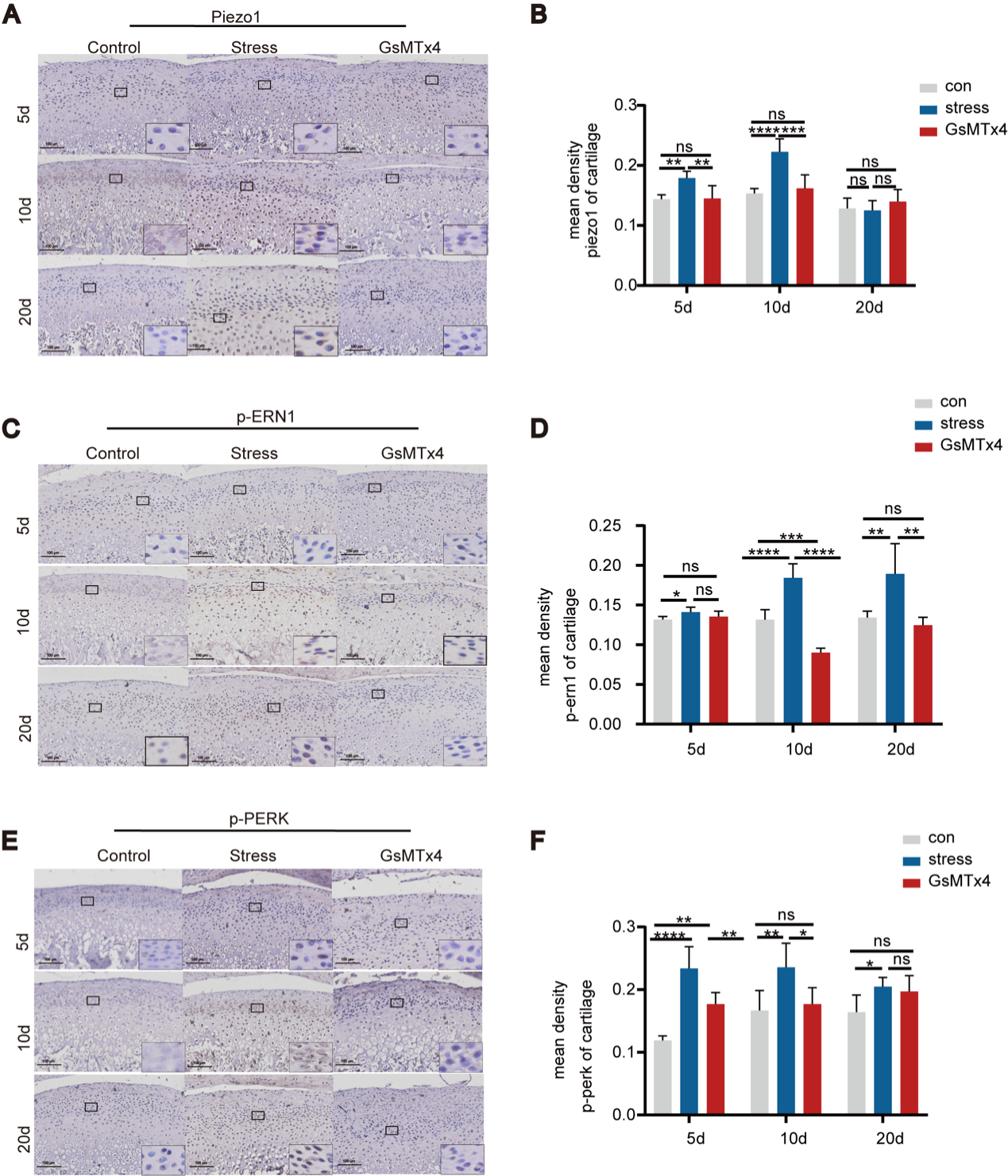
**

**Figure S7** (A and B) Piezo1 expression was evaluated by immunohistochemistry. (C-F) p-PERK and p-ERN1 expression evaluated by immunohistochemistry. All data are expressed as the mean±SEM (n=6). The results were analyzed by one-way ANOVA followed by Tukey’s test. ∗ P<0.05, ∗∗P<0.01, and∗∗∗P<0.001. Control: negative control group. Stress: forced mouth-opening model group. GsMTx4: treatment group.
